# The Epidemiological Surveillance of Mesothelioma Mortality in Italy as a Tool for the Prevention of Asbestos Exposure

**DOI:** 10.3390/ijerph20115957

**Published:** 2023-05-25

**Authors:** Lucia Fazzo, Giada Minelli, Marco De Santis, Emiliano Ceccarelli, Ivano Iavarone, Amerigo Zona

**Affiliations:** 1 Department of Environment and Health, Istituto Superiore di Sanità, 00161 Rome, Italy; marco.desantis@iss.it (M.D.S.); ivano.iavarone@iss.it (I.I.); amerigo.zona@iss.it (A.Z.); 2 Statistical Service, Istituto Superiore di Sanità, 00161 Rome, Italy; giada.minelli@iss.it (G.M.); emiliano.ceccarelli@iss.it (E.C.)

**Keywords:** malignant mesothelioma, mortality, childhood exposure, asbestos, occupational exposure, environmental exposure

## Abstract

As part of a surveillance plan active since the early 1990s, this study evaluates malignant mesothelioma (MM) mortality for the time-window 2010–2019 in Italy, a country that banned asbestos in 1992. National and regional mortality rates for MM, and municipal standardized mortality ratios (all mesotheliomas, pleural (MPM) and peritoneal (MPeM)), by gender and age group were calculated. A municipal clustering analysis was also performed. There were 15,446 deaths from MM (11,161 males, 3.8 × 100,000; 4285 females, 1.1 × 100,000), of which 12,496 were MPM and 661 were MPeM. In the study period, 266 people ≤50 years died from MM. A slightly decreasing rate among males since 2014 was observed. The areas at major risk hosted asbestos-cement plants, asbestos mines (chrysotile in Balangero), shipyards, petrochemical and chemical plants, and refineries. Female mortality excesses particularly were found in municipalities with a fluoro-edenite-contaminated mine (Biancavilla) and textile facilities. Excesses were also found in a region with the presence of natural asbestos fibres and in males living in two small islands. The Italian National Prevention Plan stated recommendations to eliminate asbestos exposures and to implement health surveillance and healthcare for people exposed to asbestos.

## 1. Introduction

All types of asbestos are certain carcinogens for humans, causing malignant mesothelioma, including cancers of the lungs, larynx and ovary; a positive association between asbestos and pharynx, stomach and colorectal cancers has also been reported [1]. Asbestos exposure also causes benign diseases: pleural plaques and fibrosis and asbestosis. Asbestos is among the main worldwide occupational carcinogens [2] and exceeding mortality from asbestos-related diseases (ARDs) among non-occupational exposed people has also been reported [3,4].

A recent review highlighted the ongoing presence of a worldwide asbestos problem in the countries where the use and production of asbestos is allowed and in asbestos-banned countries in relation to the management of residual asbestos-containing materials [5].

In 2007, the World Health Organization (WHO) and the International Labour Organization (ILO) recommended the adoption of National Asbestos Programs (NAP) [2]; recently, Arachi et al., 2021 analysed their implementation in 195 countries [6]. The countries were considered to have a “bona fide NAP” if a single document describing the national situation of asbestos and ARDs in adherence to the format published by the WHO/ILO was available. A bona fide NAP was detected in 7% of the countries, including countries in which asbestos was banned and not banned. Italy was categorized in the B-category (98 countries), having information and resources to implement a bona fide NAP [6].

The implementation of surveillance plans of ARDs, in order to eliminate them by 2023, was recommended by the WHO [7].

Deaths from MM were often used to quantify the global impact of ARDs [8].

The issue concerning the worldwide availability and reliability of MM mortality data was recently raised [9]. Odgerel et al. showed that 78% of WHO-reported MM deaths were from high-income and upper-middle-income countries, while the remaining were from low-income countries. Only 14% of middle- and low-income (MLI) countries reported MM mortality data to the WHO vs. 68% of high-income (HI) countries. This could determine a low public awareness of the health risk of asbestos and delay the ban of its use. HI countries might share technologies and information with MLI countries to detect MM cases and, primarily, to eliminate asbestos [9].

A variety of algorithms have been used to estimate the global numbers of MM deaths [9]. Based on the WHO Mortality database, the global MM burden from 1980 to 2017 was estimated in 38,400 and 30,000 annual deaths by Odgerel and the GBD, respectively [10,11]. Globocan 2018, as part of the IARC’s Global Cancer Observatory, reported 25,576 MM deaths in 130 United Nation Member States [12]. Recently, the GBD estimated that 29,251 subjects died from MM in 2019, a 0.4 (95% CI: 0.3–0.4) standardized rate × 100,000 inhabitants, considering 204 countries and territories [13].

The research on the health impact of asbestos addressed mainly occupational exposure. However, exposure to asbestos poses a risk also in the residential and domestic contexts [14,15,16,17,18,19], with cohort studies reporting excesses of early mortality from MM in people exposed to asbestos in childhood [20,21,22,23,24,25]. A residential cohort study of former school children, attending four schools located close to a large asbestos-cement plant (1928–1984, Aalborg, Denmark), showed that environmental asbestos exposure in childhood, may also increase the overall cancer risk later in life (lung, larynx and corpus uteri) [23,24,25].

Italy was one of the main producers of asbestos in Europe, and the asbestos ban occurred in 1992. A total of 4400 subjects were estimated to die each year from ARDs in the 2010–2016 period: 1515 observed deaths from MM and 58 from asbestosis were added to the estimated deaths from asbestos-related lung cancer (2830) and ovary cancer (16); these latter two were estimated based on Italian-population-based case–control studies and asbestos-cement and glasswork occupational cohorts, respectively. A possible underestimation was highlighted by the authors, considering the limited occupational sectors included in the estimates of ovarian cancers and the lack of larynx cancer deaths [26]. An investigation of MM deaths in people ≤50 years old, as an indicator of possible non-occupational asbestos exposure, estimated 487 people deceased from MM in the 2003–2016 period [27].

Since 2002 a National Registry of MM cases (ReNaM) has been operated in Italy through Regional Operating Centres (COR-ReNaM). The most recent report revealed 31,572 cases of MM diagnosed in the 1993–2018 period: 93% MM of pleura (MPM) and around 6% of peritoneum (MPeM). The average age at diagnosis was 70 years with a male-to-female ratio of 2.6:1. Of those cases with known asbestos exposure, 68% had been exposed to asbestos in the workplace, and 4% reported an environmental exposure. Building, engineering, textile and shipyard work sectors are those most involved [3].

The aim of this survey is to present an update of the rates of mortality caused by MM at the municipal level (2010–2019) in Italy, as part of a surveillance plan started in the early 1990s.

## 2. Materials and Methods

The cause-specific mortality database, managed by the Statistical Service of the Italian National Institute for Health and derived from the National Register of Causes of Death (RCoD), was analysed. The RCoD data include the underlying cause of death (i.e., the disease or injury that initiated the chain of events leading directly to death) classified according to the International Classification of Diseases, 10th revision, ICD-10. Deaths that occurred in the 2010–2019 period due to all malignant mesotheliomas (MM: ICD 10: C45) were identified, considering also the specific anatomic sites: mesothelioma of pleura (MPM: C45.0), peritoneum (MPeM: C45.1), pericardium (C45.2), other sites (C45.7) and unspecified sites (C45.9).

Mortality standardized rates (direct method, European population 2013 as reference), with 90% confidence intervals (90% CIs) by gender, age-class and anatomic site, at national level and for each of the 21 Italian regions and autonomous provinces, were computed. Annual temporal trends of all MM and MPM mortality at national level are reported.

For each of the 7903 Italian municipalities, the standardized mortality ratios (SMRs), with 90% CIs, were calculated from all MM, MPM and MPeM cases, using the regional age-class and gender-specific rates as reference. The 90% CIs were estimated based on Poisson’s distribution if the observed cases were less than 100; otherwise, the Byar method was used. The SMRs were computed in general population (all age-classes) and, for all MM cases (C45), in ≤50 years age-class, using SAS software (version 9.4).

In addition, we carried out a clustering analysis at municipal level of mortality from MM in order to identify the areas with major departures from expected figures: the clustering analysis, even if less specific with respect to SMR analysis, should be less affected by random variation due to the low number of cases. Considering the spatial distribution of all-cause and MM mortality, the country was divided into six macroareas: northeast, northwest, centre, south and the two main islands (Sicily and Sardinia) separately. These areas also correspond to the geographical distribution of the main historic industrial settings, with a higher presence in northern regions. Spatial Scan Statistic procedure was applied, using SatScan software (version 10.1). A Poisson model for the case distribution in each municipality, identified by the x, y coordinates of municipal town hall, was assumed; the maximum radius of the circular window was fixed from 0 to 50% of population and to 10 km. Clusters of interest are selected on the basis of the *p*-value, which is associated with their likelihood under the null hypothesis (*p* < 0.10).

## 3. Results

### 3.1. All-Ages Population

In Italy, in the 2010–2019 period, 15,446 subject died from MM, corresponding to about 1545/year. The deaths included 11,161 males (3.82 × 100,000 inhabitants) and 4285 females (1.10 × 100,000 inhabitants). The majority of them died from MPM (81% in males and 79% in females); 12 and 13% of all MM cases, in males and females, respectively, were reported in unspecified sites. The male/female mortality standardized rate (SR) ratio is 3.5 for all MM and 2.0 for MPeM. Table 1 reports the mortality rates from MM by anatomic site and gender.

Table 2 shows the mortality from all MM, by age-class and gender. The highest rates concern the oldest subpopulation (80+ years), which have, in both genders, a three-fold higher rate than those observed in the middle age-class (51–79 years). Mortality rates in ≤50 years age-class are, as expected, very low, considering the long latency of MM. The male/female SR ratio of mortality from all MM is lower (2.4) in the young sub-population (≤50 years) than in all ages.

**Figure 1:**
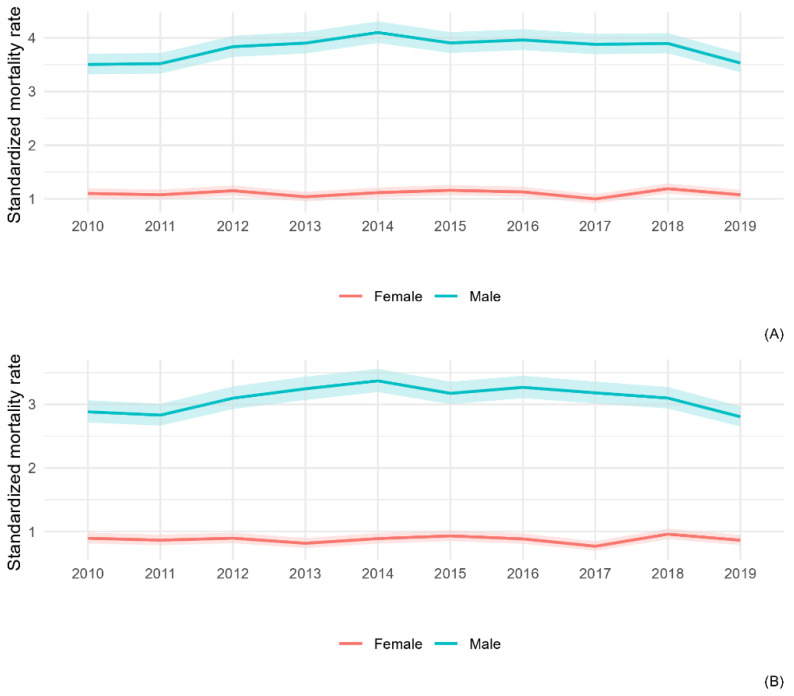
Annual temporal trend of mortality from all malignant mesothelioma (**A**) and pleural mesothelioma (**B**), by gender, from the 2010–2019 period.

The annual temporal trend of national mortality from all MM (C45) and MPM (C45.0) in the ten-year period is reported in Figure 1. Since 2014, a slight decrease in the male population is observed; among females, a peak is observed in 2018.

**Figure 2:**
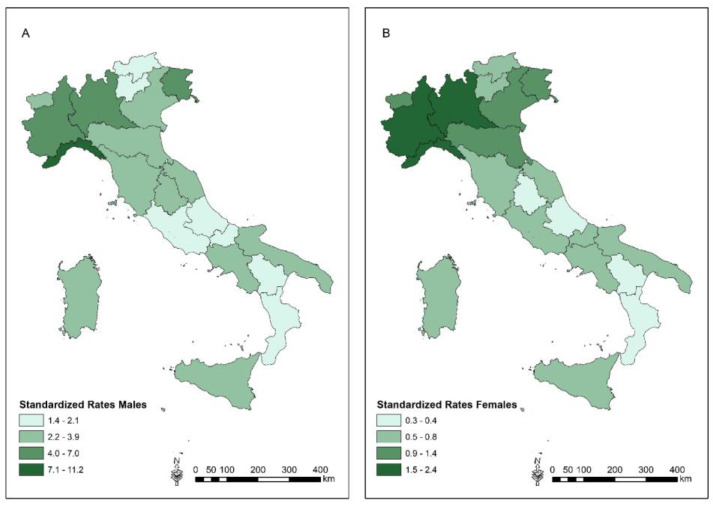
Mortality from all malignant mesothelioma (C45): regional standardized rates (×100,000 inhabitants) in males (**A**) and in females (**B**) from the 2010–2019 period.

Figure 2 shows the distribution of the SR of mortality from MM (C45) at regional level. The regional SRs that were higher than the national SRs (3.82 in males and 1.1 in females × 100,000 inhabitants) were detected in six regions for the male population (Liguria, Friuli Venezia Giulia, Piedmont, Lombardy, Aosta Valley and Emilia Romagna) and in four regions among females (Piedmont, Lombardy, Liguria and Aosta Valley). A north–south gradient is observed in both genders.

At the municipal level, statistically significant exceeding SMRs, based on at least five cases, for all MM cases were found in 170 municipalities in males and 56 municipalities in females; for MPM, 151 and 40 municipalities presented statistically significant exceeding SMRs in males and females, respectively. The analyses performed on MPeM mortality showed two municipalities with excesses (statistically significant SMR) among males (Casale Monferrato in Piedmont and Forlì in Emilia Romagna) and two among females (Grugliasco in Piedmont and Brescia in Lombardy).

**Figure 3:**
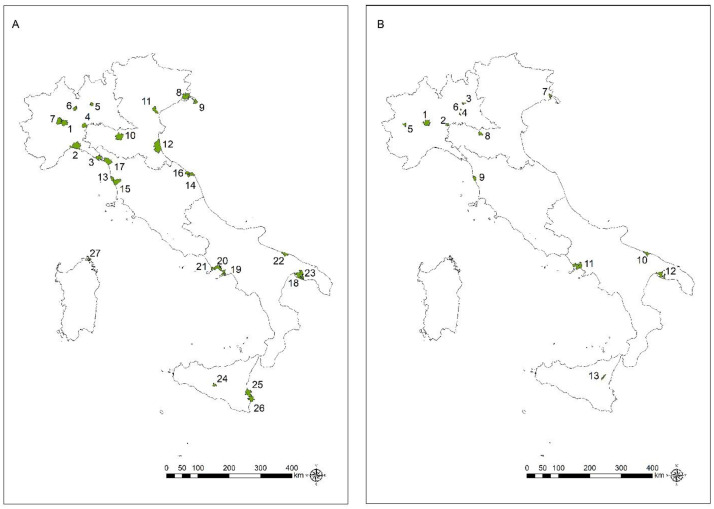
Mortality from all malignant mesothelioma (C45): significant clusters among males (**A**) and among females (**B**) from the 2010–2019 period.

**Figure 4:**
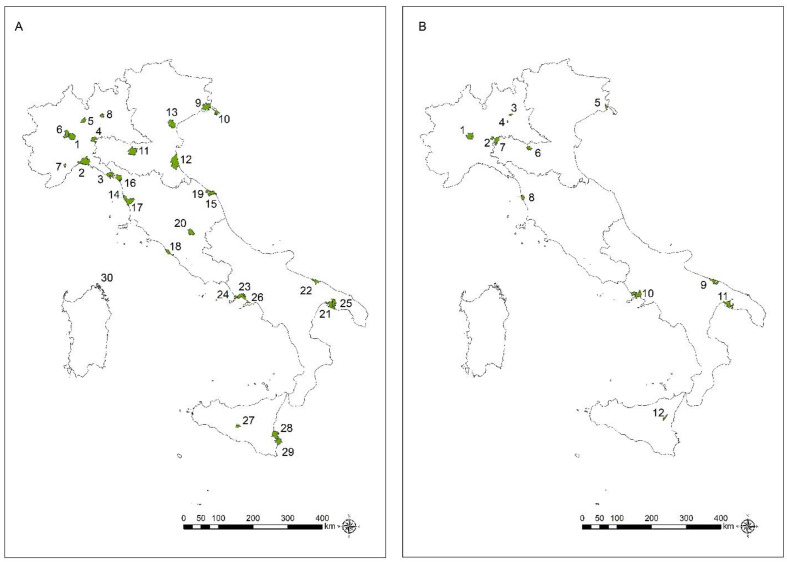
Mortality from malignant pleural mesothelioma (C45.0): significant clusters among males (**A**) and females (**B**) from the 2010–2019 period.

**Figure 5:**
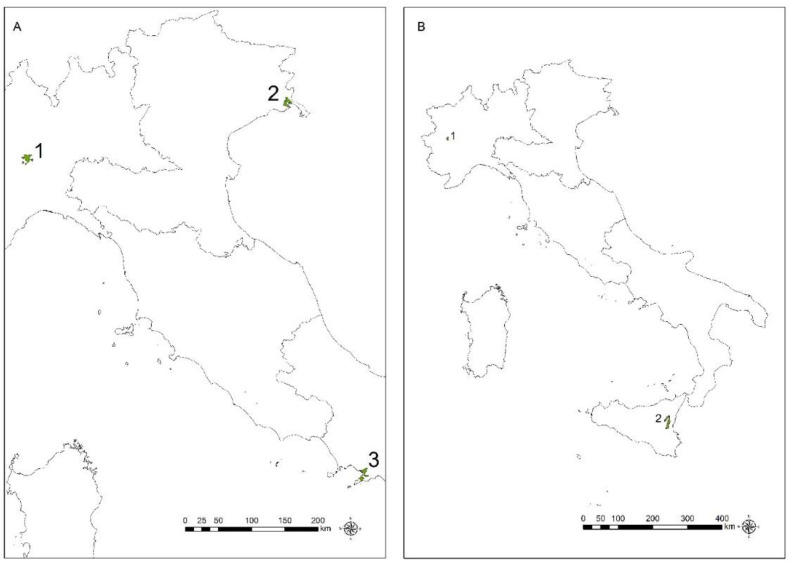
Mortality from malignant peritoneal mesothelioma (C45.1): significant clusters among males (**A**) and females (**B**) from the 2010–2019 period.

Significant clusters of mortality from MM, MPM and MPeM in the two genders separately are shown in Figure 3, Figure 4 and Figure 5, respectively. The municipalities constituting each cluster and the characteristics of the clusters are described in the Supplementary Materials (Tables S1–S6).

The areas with significant clusters for C45 (all MM) and C45.0 (MPM) match fairly closely. Statistically significant clusters for MPeM (C45.1) were detected in three areas for males (Casale Monferrato, with a former asbestos-cement plant; Monfalcone, with shipbuilding; and Torre Annunziata, also with shipbuilding) and in two areas for females, corresponding to textile facilities (Grugliasco) and a fluoro-edenite mine (Biancavilla). In these latter areas, exceeding clusters among males were not observed.

### 3.2. Focus in ≤50 Years Age-Class

Table 3 shows the standardized mortality rates in the ≤50 years age-class from all MM, MPM and MPeM cases. In the ten-year period (2010–2019), 266 people ≤50 years old died from MM: 176 males (0.12 × 100,000 inhabitants) and 90 females (0.05 × 100,000 inhabitants). They represent 1.6% (males) and 2% (females) of all deaths from MM. Around 70% in males and 60% in females of the deaths in the ≤50 years age-class from MM are due to mesothelioma of pleura, which is similar to the percentages observed in the all-ages population (about 80% in both genders, see Table 1). Conversely, the percentage of MPeM, with respect to all MM deaths, is higher in ≤50 years old people than in all ages among both sexes (16% vs. 4% in males and 20% vs. 6% in females).

**Figure 6:**
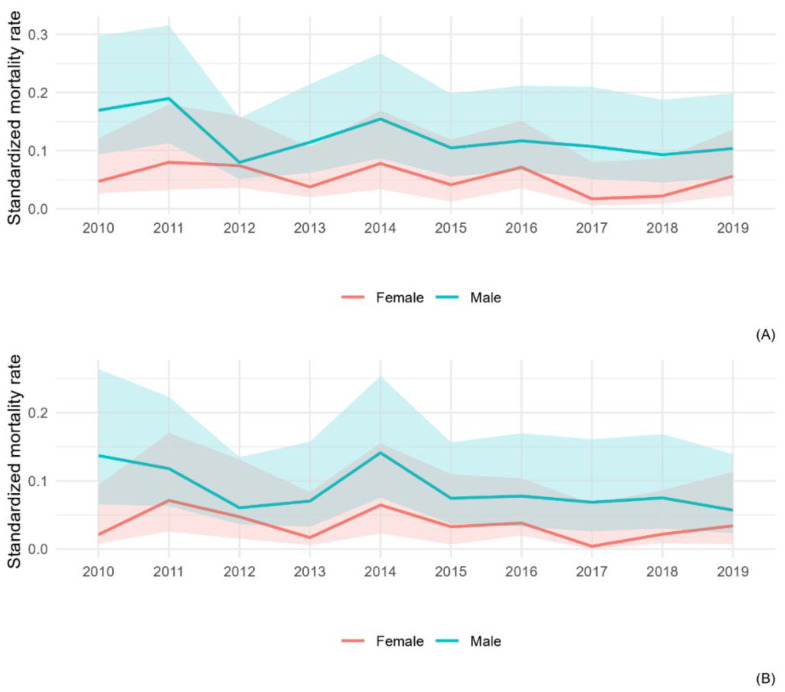
Annual temporal trend of mortality from all malignant mesothelioma (**A**) and pleural mesothelioma (**B**) in ≤50 years age-class, by gender, from the 2010–2019 period.

Figure 6 shows the annual temporal trend of MM and MPM, in this age-class, in the ten-year period. Among males, a slightly decreasing trend is observed in mortality from MPM since 2015, which is less noticeable than that from all MM observed in the all-ages male population.

**Figure 7:**
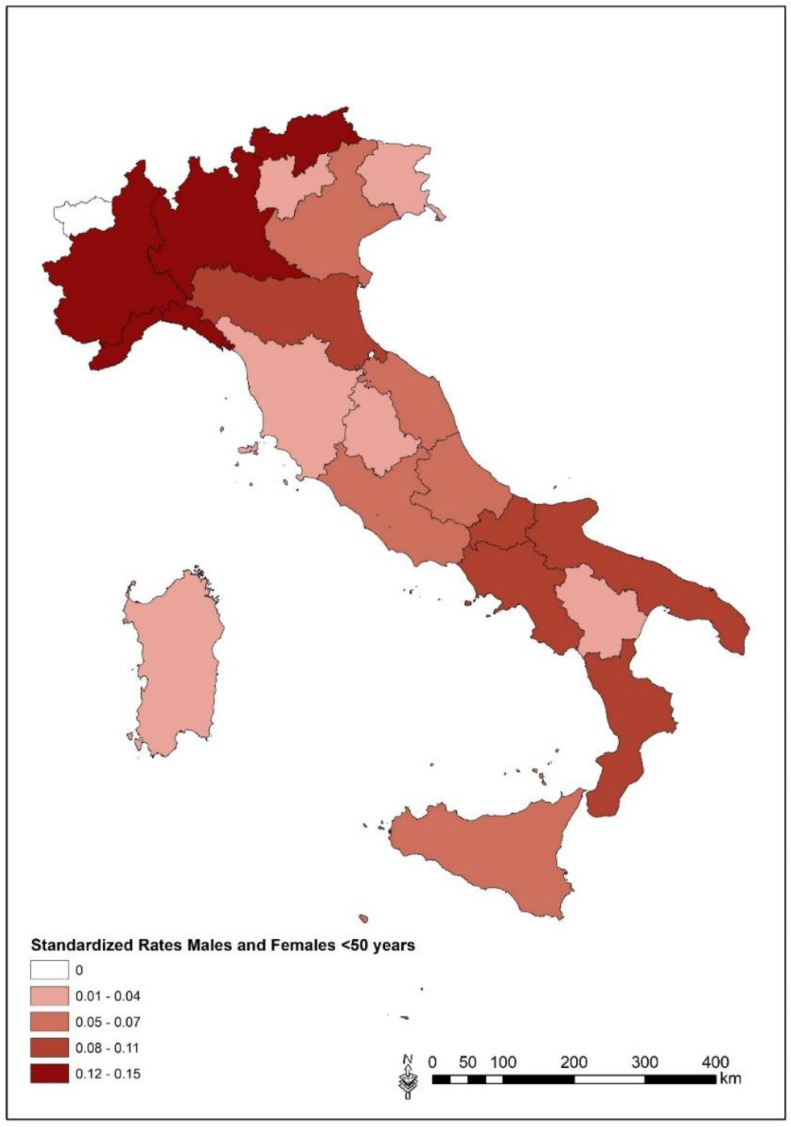
Mortality from all malignant mesothelioma (C45) in ≤50 years age-class. Regional standardized rates (×100,000 inhabitants) of males and females together from the 2010–2019 period.

Figure 7 shows the geographical distribution of standardized mortality rates in the ≤50 years age-class, by region, in the period 2010–2019. The early mortality (≤50 yrs) from MM at the regional level compared to the national rates shows a higher risk in some Northern Italian regions (Piedmont and Lombardy) and Molise, which display significantly higher SMRs than expected. Liguria, Veneto, Bolzano and Trento Provinces, Emilia Romagna and Marche show risks that are higher than the national risk.

In the analysis at municipal level, 212 out of the 7903 Italian municipalities showed at least one MM death at early ages (≤50 years) in the study period. Exceeding mortality from MM was observed in 205 municipalities, and in 169 of them, the excess risk was statistically significant. A statistically significant exceeding risk, based on at least five cases (males and females combined), was detected in Casale Monferrato (SMR 2878.8, 90% CI 1622.1–5109.0), while non-significant excesses were observed in Naples (SMR 170.8, 90% CI 83.2–351) and Milan (SMR 114.5, 90% CI 68.4–191.5).

## 4. Discussion

In Italy, from 2010 to 2019, 15,446 subjects died from MM (11,161 males and 4285 females), corresponding to a rate ×100,000 inhabitants of 3.8 in males and 1.1 in females. Out of them, 12,496 MPM and 661 MPeM deaths were detected.

In the previous update of the Italian MM mortality surveillance plan (2003–2014) about 1340 deaths/year were estimated: in the entire period, 16,086 MM deaths, including 13,051 MPM and 747 MPeM, were retrieved [28]. The results of the present update, corresponding to about 1546 deaths/year, confirm that Italy is among the countries most impacted by ARDs, in terms of mortality: the GBD estimated 29 thousand annual deaths from MM, corresponding to 0.4 × 100,000 inhabitants, in 204 countries from 2010 to 2019 [13]. The possible GBD underestimation of the global burden of MM due to underreporting by LMI countries, though a possible overestimation for HI incomes, was recently highlighted [9]. The annual average number of the observed MM deaths reported in the present paper is in agreement with the 2019 GBD estimations for Italy, with SR differences due to the methods used in their computation (https://vizhub.healthdata.org/gbd-compare, accessed on 17 May 2023).

In around 80% of observed MM deaths, mesotheliomas are localized in pleura (12,496), and 661 deaths involve mesotheliomas in the peritoneum. A range of 12–13% of deaths classified as MM are in unspecified sites, and, considering the distribution of MM by anatomic site, we could hypothesize an underestimation of MPM. MPeM is rarer, representing 4.3% of all MM, with a M/F rate lower than that of all MM (2.0 in MPeM vs. 3.5 in all MM). Considering that MPeM is caused by high levels of asbestos exposure, occurring usually in occupational contexts [29], a major potential misclassification, in females compared to males, for ovarian cancer and other abdomen contiguous tumours was hypothesized among the causes of the lower M/F rate for MPeM [30]. The highest rates of MM mortality were observed in the oldest age-class (+80 years), even if the majority of observed cases concern the 51–79 age-class. The high percentage of MM in unspecified sites is likely to be associated with the low diagnostic certainty reported, particularly in the oldest people [3]. The annual temporal trend shows a slight decrease among males since 2014; among females, a peak is observed in 2018. The temporal trend in the ≤50 years age-class is less clear because of the low number of deaths (wide CIs), even if the slight decrease since 2014 in men is also observed in this age-class. The forecast of MPM mortality in Italy by 2040, based on the asbestos consumption and male MPM mortality in the 1970–2014 period, predicted the peak of MPM deaths to occur in 2021, with a subsequent decrease (1122 MPM deaths in 2021 and 344 in 2039) [31].

In Italy, the use/production/import and export of asbestos were banned in 1992. Han 2022 supposed that MM cases might begin to decrease after two decades of a complete ban of asbestos use, observing the greatest decreases in the countries with the highest socio-demographic indexes, which had a large industrial use of asbestos in the past and implemented limitations and bans earlier [32]. In addition to the 30–40 years of the MM latency period [6], the residual asbestos exposure contexts could also imply that it might take a long time to observe the decrease in incident cases after the asbestos ban [32]. The exposure–response curve elaborated by the model based on asbestos consumption and male MPM mortality in Italy suggested that the most relevant contributions to the risk come from exposure occurring 20–40 years before death [31]. Thives and colleagues reported that the pathogenic effects of asbestos, including asbestosis, pleural plaques and neoplasms, can appear even 60 years after exposure [5].

The regional distribution observed in the present investigation confirmed the geographical north–south gradient reported in the previous periods, in agreement with the historical industrialization of the country, since the early 1900s. Four regions presented higher rates than the national rate in both genders: Piedmont, Lombardy, Liguria and Aosta Valley. In Piedmont and Lombardy, two asbestos-cement plants operated in the past (in Casale Monferrato and Broni, respectively), and epidemiological studies reported a high risk of MM mortality for former workers, their relatives and the population living near those asbestos-cement plants [33,34]. In addition, in Piedmont, a chrysotile mine (Balangero) was active, and a high risk of MM in the occupational cohort was reported [35]. Lombardy is an industrialized region with a high presence of facilities, including the above-cited asbestos-cement plant in Broni, and the presence of the talc-mine in Valmalenco is reported among the potential asbestos contamination sources in the Seventh Report of ReNaM [3]. Additionally, a study based on COR-ReNaM data suggested asbestos exposure opportunities in agricultural settings [36]. Liguria is a coastal region with petrochemical/refineries and shipbuilding activities, both of which are occupational settings where asbestos exposure due to the presence of asbestos-containing materials (insulation) were reported [3,37]. The high rates observed in Aosta Valley deserve specific mention: the region is located in the Western Alps, and the presence of chrysotile and tremolite asbestos minerals was documented in mines of Crètaz and Emarese, which are among the main Italian mines operated since 1979 and 1968, respectively [38]. A high regional mortality rate was observed for the first time in the 2004–2013 period without observed municipal excesses [28]. In the present investigation, an exceeding SMR was observed in the Aosta municipality, even if not statistically significant, based on nine male MM deaths (SMR: 120, 90% CI: 70–206). MM deaths in the ≤50 years age-class were not observed in Aosta Valley. The Seventh ReNaM Report stated 59 incident MM cases in the 2000–2018 period, based on the Aosta Valley COR-ReNaM, and highlighted the presence of natural fibres of serpentine asbestos in the rocks and, for economic activities, a steel industry and a former mine of chrysotile [3]. Emarese, a municipality where a mine operated, has been included among the contaminated sites of national concern for remediation since 2006. The results of the present investigation (no deaths from MM) in Emarese are in agreement with the recent report of the Italian National Epidemiological Surveillance Programme of contaminated sites [39].

The analyses at municipal level showed a higher number of statistically significant SMRs for all MM, with respect to MPM, confirming the need to detect all MMs.

Cluster analysis, compared to SMRs, is more suitable to detect specific territories with the highest risks of MM mortality. As expected, considering that the main sources of asbestos exposure occurred in occupational settings, where, in Italy, the male workforce is historically preeminent, the highest number of exceeding significant clusters are found among males.

The clusters for MM and MPM are often overlapping. Statistically significant clusters among the male population are observed in the areas with known asbestos exposure sources, confirming the persistence of asbestos health impact. These areas include harbours with shipbuilding (Castellammare, Pozzuoli and Monfalcone) and several kinds of facilities, such as petrochemical/refineries and steel plants (i.e., Genoa, La Spezia, Trieste, Ravenna, Livorno, Ancona, Civitavecchia, Naples, Taranto, Falconara and Augusta), and areas close to former asbestos-cement plants (Casale Monferrato, Broni, Bari, San Cataldo and Syracuse). In addition, significant clusters were observed near operating steel plants (Terni and Dalmine) and chemical facilities (Mira, Cengio and Rosignano Marittima).

Recently, a review highlighted the risk of MM in sailors, including military servicemen [40]. The widespread presence of asbestos fibres in confined spaces in vessels was documented [3,40]. In the present investigation, the possible role of asbestos exposure in this occupational setting is conceivable for the exceeding clusters found in harbours and, in particular, in La Maddalena, an island close to Sardinia where an Italian Navy shipyard is located, and in Procida, a small island in the Gulf of Naples, where a high percentage of the male population is employed as sailors in merchant ships.

The excesses observed in female populations could be of particular concern as an indicator of possible non-occupational asbestos exposures. In females, significant clusters for MM and MPM were found in some of the areas showing high risks in male populations. The risks for wives and cohabitants of former workers and in people living close to the asbestos-cement plants were reported in cohort studies performed in Casale Monferrato [41]. A recent study, based on individual data and information from the Apulia COR-ReNaM, was performed in Bari, considering the MM incident cases (1988–2019 period) with residential asbestos exposure alone, and it reported a risk in the population living near the asbestos-cement plant, confirming the previous analyses [42]. In Naples, a large Southern municipality, there are several potential sources of occupational and non-occupational asbestos exposure, i.e., harbours, industrial areas and former asbestos-cement plant: excesses of MM were reported in the former workers of the asbestos-cement plant [43] and in people living in the neighbourhoods near the plant [44]. A risk related to the use of recycled jute sacks previously containing asbestos, in an area close to the harbour, was also reported [45]. In addition, the “urban” asbestos risk, due, in particular, to the presence of asbestos-containing materials in buildings and to traffic, could be considered in large urban areas [46,47,48,49]. Excesses in females were observed also in areas corresponding to huge harbours, including petrochemical plants and refineries (Livorno and Taranto).

Three areas presented statistically significant increased clusters of MM and MPM in females only. In Sarnico (Lombardy) and Grugliasco (Piedmont) non-asbestos textile facilities are located, and the main workforce was historically constituted by females; the risk of MM incidence and mortality among these female workers’ cohorts was documented: the peculiarity of the Sarnico factory was also its location near an asbestos textile plant that shared the neighbouring area [15,50]. Biancavilla is a town localized at Etna Volcano slopes, and the presence of naturally occurring fluoro-edenitic fibres in a stone quarry was documented: the cancerogenicity of fluoro-edenite was also defined considering the epidemiological studies performed in Biancavilla and in vivo and in vitro studies [51]. In the area of another cluster in Lombardy (Romanengo and Cumignano sul Naviglio), a former asbestos fibre textiles plant was located (Romanengo).

Statistically significant clusters for MPeM are rarer, as expected, considering the high levels of asbestos exposure causing the disease. Among males, exceeding clusters of MPeM mortality were shown in areas close to the former asbestos-cement plant of Casale Monferrato and shipyards in Monfalcone and Torre Annunziata. Among females, significant clusters of MPeM mortality were found in the areas with textile facilities in Grugliasco, and in Biancavilla, where the widespread contamination by natural fluoro-edenitic fibres and a high risk of MM were documented [51,52,53,54,55].

Mortality rates in the ≤50 years age-class are, as expected, very low, but early deaths from MM represent a proxy of exposure in childhood due to the long latency of MM and the possible etiologic role of environmental asbestos exposure in childhood. The male/female SR ratio is lower in the young sub-population (≤50 years) compared to the all-ages population (2.4 vs. 3.5). Most MM deaths in this age-class refer to pleural MM (about 70% in men and 60% in women); however, these percentages are lower than those observed in all ages (about 80% in both genders). Conversely, the percentage of MPeM, with respect to all MM deaths, is higher in ≤50 years old people than in all ages, among both sexes (16% vs. 4% in men and 33% vs. 6% in women). This observation is in agreement with the distribution of MPeM and MM cases by age-class, as reported in the latest ReNaM Report [3]. Moreover, this finding could be attributed, in part, to a higher quality of diagnostic ascertainment in young cases, as stated by the latest ReNaM Report [3]. The low M/F rate and the young age of deaths, considering the latency period, push the hypothesis of non-occupational asbestos exposure in these subjects. The slightly slower decrease in MPM mortality in the ten-year period starting in 2014 observed in the ≤50 years age-class reflects the annual temporal trend observed in the all-ages population, but the low number of deaths in this age-class limits a clear interpretation.

As for all ages, a significant excess of mortality among people ≤50 years old was observed in Piedmont (SMR: 200, 90% CI: 127–313; 35 deaths) and in Lombardy (SMR: 153, 90% CI: 108–215; 65 deaths), while a non-significant excess was observed in Liguria (SMR: 130, 90% CI: 52–324; 13 deaths). The excesses (with at least five cases) were associated with the municipalities of Casale Monferrato in Piedmont, where a former asbestos-cement plant was operated, and in the large municipalities of Milan and Naples, where “urban” asbestos exposure, in addition to the asbestos sources abovementioned, may be hypothesized [46,47,48].

The limitations of MM mortality data, with respect to the incidence data, and the need to integrate the MM mortality database and the MM incident cases registry were previously discussed [26,28,56]. The present investigation highlights areas of the national territory with a high risk of MM mortality and, consequently, of past or current asbestos exposure sources. These areas, as discussed above, are often characterized by the presence of activities reported among the main asbestos exposure contexts in the latest ReNaM Report, based on individual incident case information. The agreement between the two databases, also considering the differences due to their characteristics, confirms the usefulness of MM mortality surveillance to detect the areas at major risk of asbestos exposure. This approach could be of particular interest for countries where a registry of MM incident cases is not yet operating or in the sub-areas with a limited detection of MM incident cases.

MM cases retrieved from mortality and incidence registries, as described in the Introduction, are often used to track asbestos risk contexts and represent useful indicators of asbestos exposures, but the health impact of asbestos also includes other ARD cases (asbestosis and lung, ovary and larynx cancers). The variability of overall ARD estimates could be very large, particularly for the fractions attributable to asbestos of the diseases at low etiological fractions (lung, ovarian and larynx cancers), because of the methods used. In Italy, the estimates of deaths from ARDs by year range from 4400 [26] to 14,000 (https://vizhub.healthdata.org/gbd-compare, accessed on 17 May 2023): the first was based on the risks computed in Italian contexts, and the GBD’s applied algorithms and methods at global level. Public health programs should therefore take care of all ARD cases (deaths and incident cases) to assess adequate social and health prevention and assistance actions.

In Italy, the long period of MM mortality surveillance on the overall national territory allowed the implementation of public health programs, even if much remains to be done, as highlighted by the high risks reported in the present study. The results of the national epidemiological surveillance plans and the analytic epidemiological studies performed since the 1980s in some asbestos-exposed cohorts contributed to an increase in the awareness of the asbestos impact in populations, leading administrators to enact the asbestos ban law in 1992 (Law n.257/1992. Available online: www.gazzettaufficiale.it/eli/id/1992/04/13/092G0295/sg, accessed on 17 May 2023). The epidemiological studies performed in Biancavilla, following the first signals of mortality surveillance, contributed to the definition of the cancerogenicity of fluoro-edenitic fibres [51].

Epidemiological studies contributed to the inclusion of several areas with a high risk of MM within the contaminated sites at national concern for remediation (by 1996 law) because of the presence of asbestos sources, i.e., Biancavilla, San Filippo del Mela in the Milazzo area, Casale Monferrato, Bari, Balangero, Broni and Emarese. In Casale Monferrato, the area of the former asbestos-cement plant is currently an urban park, and in Biancavilla, the environmental remediation acts concerning the former mine started recently (at the end of February 2023). The remediation of the area of the former asbestos-cement plant in Bari was completed in 2018 [42], whereas for the mine of Balangero and the asbestos-plant in Broni, the clean-up actions are still ongoing [57,58,59].

The 2020–2025 Italian National Prevention Plan (NPP) provides the implementation of knowledge of the environmental and health impacts of asbestos and the elimination of asbestos exposures, including the risks related to asbestos’ indirect use. The NPP states recommendations for environmental remediation, including guidelines for asbestos-containing material disposal, and for occupational exposure elimination. The NPP envisages the implementation and evaluation of the regional health surveillance and counselling plans to address to people former and currently exposed to asbestos; the plans have been required by law since 2008 (D.Lgs 81/2008. Available online: www.gazzettaufficiale.it/eli/id/2008/04/30/008G0104/sg, accessed on 17 May 2023), but their implementation is still fragmentary and is not homogeneous across the country. In this regard, we point out the excesses found in the San Cataldo (Sicily) municipality, where an asbestos-cement plant was operated. The cohort of the ex-workers has not been investigated and has not been included in meta-analytic studies on Italian workers exposed to asbestos [60]. In addition, these workers have not been taken into account by the Sicilian Regional health surveillance plan, which currently concerns San Filippo del Mela and Biancavilla.

The highlighted MM risks in subjects environmentally exposed to asbestos, particularly in recent years, increased the public awareness about the effect of environmental asbestos exposures and pushed out the inclusion of subjects “environmentally” exposed in compensation acts and in some regional surveillance plans. In Italy, 4% of MM cases with known asbestos exposure sources were reported due to environmental exposures [3], and MM risks were documented in populations living close to asbestos-cement plants [20,34,44] and quarries containing chrysotile and fluoro-edenitic fibres [61,62]. In addition, some areas were highlighted because of the presence of both naturally asbestos fibres and MM risks: serpentine and metabasite outcrops in Pollino Mount (Basilicata Region) [63], the abovementioned areas in Aosta Valley [38] and tremolite in Upper Susa Valley in Piedmont [64]. A recent paper highlighted the risk of MM in the areas with ophiolite minerals in Calabria [65], and the contamination of asbestos fibres in ophiolitic rocks in Emilia Romagna was stated in the Seventh Report of ReNaM among the potential asbestos exposure sources [3].

Finally, the results confirm that in Italy asbestos still represents a health problem even after its ban in 1992. A slight decrease seems to appear only in the last years. This consideration assumes further relevance considering that exceeding mortality risks are also observed among people aged less than 50 years and among females, suggesting an undeniable role of non-occupational exposures. Specific actions and coordinated management addressed to eliminate asbestos-related diseases are needed to assure a future habitable and sustainable environment [5]. Among these actions, environmental awareness via education, joint actions by society and governmental and non-governmental organizations that ensure compliance with legislation, and the improvement of mechanisms for practical oversight [5] are, in our opinion, particularly appropriate in our country. Considering the residual presence of asbestos-containing material still in place, buildings included, interdisciplinary research to identify the impact of diseases caused by asbestos and the mapping of asbestos sources are needed in these countries [5]. In this context, the development of appropriated methods of soil remediation and asbestos disposal are required. In addition, health surveillance plans, healthcare and social assurance and welfare for the victims of asbestos, including those non-occupationally exposed, could be implemented at local and national levels. The experiences performed in the asbestos-banned countries, such as Italy, could help other countries to quantify the environment and health impact of asbestos. The availability of data on ARDs could contribute to increase the public awareness on the health risk of asbestos and push out the ban of asbestos.

## Figures and Tables

**Table 1:** Mortality from malignant mesothelioma, by anatomic site and gender. Observed cases and standardized rates (×100,000 inhabitants) from the 2010–2019 period.

Cause of Death	ICD	Males		Females	
		Cases	SR (90% CI)	Cases	SR (90% CI)
All MM	C45	11,161	3.82 (3.76–3.88)	4285	1.10 (1.07–1.13)
Pleural MM	C450	9084	3.10 (3.05–3.16)	3412	0.87 (0.85–0.90)
Peritoneal MM	C451	409	0.14 (0.13–0.15)	252	0.07 (0.06–0.08)
MM pericardium	C452	6	0.0020 (0.0009–0.0048)	3	0.00091 (0.00025–0.0026)
MM of other sites	C457	282	0.097 (0.088–0.11)	108	0.027 (0.023–0.033)
MM, unspecified	C459	1380	0.48 (0.45–0.50)	510	0.13 (0.12–0.14)

SR: standardized rates; 90% CI: 90% confidence interval.

**Table 2:** Mortality from all malignant mesothelioma (ICD 10: C45), by age-class and gender: cases and standardized rates from the 2010–2019 period.

Age-Class (Years)	Males		Females	
	Cases (Percentage of All Cases)	SR (90% CI)	Cases (Percentage of All Cases)	SR (90% CI)
≤50	176 (1.6%)	0.12 (0.10–1.15)	90 (2.1%)	0.05 (0.04–0.07)
51–79	7800 (69.9%)	7.90 (7.75–8.05)	2607 (60.8%)	2.26 (2.19–2.34)
80+	3185 (28.5%)	21.80 (21.16–22.47)	1588 (37.1%)	6.27 (6.01–6.54)
All	11,161 (100%)	3.82 (3.76–3.88)	4285 (100%)	1.10 (1.07–1.13)

SR: standardized rates; 90% CI: 90% confidence interval.

**Table 3:** Mortality from all, pleural and peritoneal malignant mesothelioma (MM) in ≤50 years age-class, by gender, from the 2010–2019 period.

Cause of Death	ICD	Males		Females	
Cause of Death	ICD	Cases	SR (90% CI)	Cases	SR (90% CI)
All MM	C45	176	0.12 (0.10–0.15)	90	0.05 (0.04–0.07)
Pleural MM	C450	123	0.088 (0.071–0.11)	54	0.03 (0.025–0.049)
Peritoneal MM	C451	29	0.017 (0.011–0.028)	18	0.0080 (0.0000–0.0051)

SR: Standardized rate; CI: confidence interval; ICD: International Classification of Diseases.

## Data Availability

The data analysed in this study are subject to the following licenses/restrictions: the analysis of the data used in this study complies with the European General Data Protection Regulation (EU GDPR 2016/679), which authorized the processing of personal data relating to causes of death by ISS and other public institutions for reasons of public interest in the field of health. Written consent for participation was not required for this study, in accordance with national legislation and institutional requirements. Requests for access to these datasets should be directed to G.M., giada.minelli@iss.it.

## Supplementary Materials

The following supporting information can be downloaded at https://www.mdpi.com/article/10.3390/ijerph20115957/s1. Table S1: Mortality for all malignant mesothelioma, among males, 2010–2019. Statistically significant clusters had a *p*-value < 0.10. Table S2: Mortality for all malignant mesothelioma, among females, 2010–2019. Statistically significant clusters had a *p*-value < 0.10. Table S3: Mortality for malignant pleural mesothelioma, among males, 2010–2019. Statistically significant clusters had a *p*-value < 0.10. Table S4: Mortality for malignant pleural mesothelioma, among females, 2010–2019. Statistically significant clusters had a *p*-value < 0.10. Table S5: Mortality for malignant mesothelioma of peritoneum, among males, 2010–2019. Statistically significant clusters had a *p*-value < 0.10. Table S6: Mortality for malignant mesothelioma of peritoneum, among females, 2010–2019. Statistically significant clusters had a *p*-value < 0.10.
