# Supplementary material for: The Epidemiological Surveillance of Mesothelioma Mortality in Italy as a Tool for the Prevention of Asbestos Exposure
Source: Int J Environ Res Public Health. 2023 May 25;20(11):5957. doi: 10.3390/ijerph20115957 (PMC10252364; doi:10.3390/ijerph20115957)
Supplement: Supplementary file 1 [file ijerph-20-05957-s001.zip › ijerph-2331161-supplementary/Table S1.pdf]

Table S1. Mortality for all malignant mesothelioma, among males, 2010-2019. Statistically significant clusters (p-value <0.10).

Area	Cluster number	Radius (km)	Number of municipalities	Observed	Expected	RR
NW	1 [a]	9.11	16	223	17.57	13.24
NW	2 [b]	9.85	11	543	230.50	2.52
NW	3 [c]	9.28	9	212	51.93	4.22
NW	4 [d]	7.53	19	86	12.72	6.86
NW	5 [e]	4.93	14	61	26.09	2.35
NW	6 [f]	6.00	13	90	47.04	1.93
NW	7 [g]	9.82	14	26	7.09	3.68
NE	8 [h]	9.27	17	129	22.04	6.14
NE	9 [i]	7.00	3	146	51.98	2.93
NE	10 [j]	9.41	5	78	39.19	2.03
NE	11 [k]	7.30	5	53	25.95	2.07
NE	12 [l]	0	1	64	34.14	1.90
C	13 [m]	0	1	94	23.56	4.18
C	14 [n]	0	1	51	14.55	3.59
C	15 [o]	9.84	4	23	6.24	3.72
C	16 [p]	7.69	6	30	9.97	3.05
C	17 [q]	9.83	7	56	27.37	2.08
S	18 [r]	0	1	81	22.02	3.84
S	19 [s]	8.34	11	68	26.99	2.60
S	20 [t]	9.64	13	211	133.14	1.68
S	21 [u]	5.77	4	41	12.69	3.30
S	22 [v]	8.31	2	77	39.44	2.01
S	23 [w]	9.49	8	29	10.76	2.73
SIC	24 [x]	0	1	18	2.62	7.04
SIC	25 [y]	8.40	3	25	7.90	3.26
SIC	26 [z]	0	1	35	15.72	2.30

SAR	27 [aa]	0	1	8	1.42	5.82

[a] Casale Monferrato, Rosignano Monferrato, San Giorgio Monferrato, Frassineto Po, Villanova Monferrato, Cella Monte, Morano sul Po, Ticineto, Balzola, Coniolo, Terruggia, Borgo San Martino, Ozzano Monferrato, Rive, Treville, Motta de' Conti.

[b] Genova, Serra Riccò, Busalla, Campomorone, Sant'Olcese, Ceranesi, Davagna, Savignone, Mignanego, Montoggio, Casella.

[c] La Spezia, Vezzano Ligure, Arcola, Lerici, Portovenere, Santo Stefano di Magra, Riomaggiore, Follo, Riccò del Golfo di Spezia.

[d] Broni, Stradella, Cigognola, Pietra de' Giorgi, Portalbera, Barbianello, Santa Giuletta, Zenevredo, Bosnasco, Campospinoso, Canneto Pavese, Casanova Lonati, Mornico Losana, Redavalle, San Cipriano Po, Albaredo Arnaboldi, Castana, Montù Beccaria, Montescano.

[e] Dalmine, Ponte San Pietro, Treviolo, Mapello, Bonate Sopra, Curno, Suisio, Bonate Sotto, Osio Sopra, Chignolo d'Isola, Filago, Terno d'Isola, Madone, Presezzo.

[f] Legnano, Parabiago, Canegrate, Busto Garolfo, Dairago, Villa Cortese, Inveruno, Ossona, Buscate, Casorezzo, Mesero, San Giorgio su Legnano, Arconate.

[g] Trino, Cerrina Monferrato, Gabiano, Mombello Monferrato, Palazzolo Vercellese, Pontestura, Tricerro, Odalengo Grande, Ronsecco, Camino, Fontanetto Po, Moncestino, Solonghello, Villamiroglio.

[h] Monfalcone, Ronchi dei Legionari, Staranzano, San Canzian d'Isonzo, Fogliano Redipuglia, Cervignano del Friuli, Aquileia, Romans d'Isonzo, Ruda, San Pier d'Isonzo, Aiello del Friuli, Fiumicello Villa Vicentina, Doberdò del Lago, Terzo d'Aquileia, Turriaco, Campolongo Tapogliano, Sagrado.

[i] Trieste, Muggia, San Dorligo della Valle.

[j] Reggio nell'Emilia, Correggio, Bagnolo in Piano, Cadelbosco di Sopra, San Martino in Rio.

[k] Spinea, Mira, Mirano, Martellago, Salzano.

[l] Ravenna.

[m] Livorno.

[n] Ancona.

[o] Rosignano Marittimo, Castellina Marittima, Chianni, Santa Luce.

[p] Falconara Marittima, Montemarciano, Agugliano, Monte San Vito, Chiaravalle, Polverigi.

[q] Carrara, Massa, Seravezza, Forte dei Marmi, Pietrasanta, Montignoso, Stazzema.

[r] Taranto.

[s] Castellammare di Stabia, Torre Annunziata, Pompei, Scafati, Gragnano, Santa Maria la Carità, Meta, Agerola, Vico Equense, Casola di Napoli, Sant'Antonio Abate.

[t] Napoli, Torre del Greco, Portici, Casalnuovo di Napoli, Ercolano, Casoria, San Giorgio a Cremano, Volla, Sant'Anastasia, Casavatore, Cercola, Massa di Somma, San Sebastiano al Vesuvio.

[u] Pozzuoli, Procida, Bacoli, Monte di Procida.

[v] Bari, Triggiano.

[w] Grottaglie, San Giorgio Ionico, Leporano, Carosino, Lizzano, Monteiasi, Pulsano, Faggiano.

[x] San Cataldo.

[y] Augusta, Melilli, Priolo Gargallo.

[z] Siracusa.

[aa] La Maddalena.
