# Supplementary material for: The Epidemiological Surveillance of Mesothelioma Mortality in Italy as a Tool for the Prevention of Asbestos Exposure
Source: Int J Environ Res Public Health. 2023 May 25;20(11):5957. doi: 10.3390/ijerph20115957 (PMC10252364; doi:10.3390/ijerph20115957)
Supplement: Supplementary file 1 [file ijerph-20-05957-s001.zip › ijerph-2331161-supplementary/Table S2.pdf]

Table S2. Mortality for all malignant mesothelioma, among females, 2010-2019. Statistically significant clusters (p-value <0.10).

Area	Cluster number	Radius (km)	Number of municipalities	Observed	Expected	RR
NW	1 [a]	9.49	14	176	9.23	20.55
NW	2 [b]	3.44	5	63	4.14	15.62
NW	3 [c]	4.56	7	22	3.46	6.41
NW	4 [d]	0	1	9	0.61	14.83
NW	5 [e]	4.45	4	48	23.08	2.10
NW	6 [f]	3.47	2	6	0.50	12.09
NE	7 [g]	5.34	4	18	3.76	4.88
NE	8 [h]	6.22	3	13	1.94	6.82
C	9 [i]	0	1	29	8.05	3.75
S	10 [j]	0	1	39	11.53	3.61
S	11 [k]	7.22	17	92	46.90	2.22
S	12 [l]	0	1	23	7.03	3.39
SIC	13 [m]	0	1	11	0.70	16.73

[a] Casale Monferrato, San Giorgio Monferrato, Ticineto, Frassineto Po, Villanova Monferrato, Occimiano, Ozzano Monferrato, Pontestura, Terruggia, Balzola, Rosignano Monferrato, Cella Monte, Motta de' Conti, Treville.

[b] Broni, Stradella, Canneto Pavese, Portalbera, Zenevredo.

[c] Sarnico, Credaro, Paratico, Predore, Villongo, Foresto Sparso, Viadanica.

[d] Calcio.

[e] Collegno, Grugliasco, Rivoli, Beinasco.

[f] Romanengo, Cumignano sul Naviglio.

[g] Monfalcone, Ronchi dei Legionari, Fogliano Redipuglia, Staranzano.

[h] Bagnolo in Piano, Castelnovo di Sotto, Cadelbosco di Sopra.

[i] Livorno.

[j] Bari.

[k] Napoli, Casoria, Giugliano in Campania, Afragola, Marano di Napoli, Volla, Caivano, Calvizzano, Frattamaggiore, Arzano, Cardito, Casalnuovo di Napoli, Casavatore, Grumo Nevano, Melito di Napoli, Sant'Antimo, Villaricca.

[l] Taranto.

[m] Biancavilla.
