# Supplementary material for: The Epidemiological Surveillance of Mesothelioma Mortality in Italy as a Tool for the Prevention of Asbestos Exposure
Source: Int J Environ Res Public Health. 2023 May 25;20(11):5957. doi: 10.3390/ijerph20115957 (PMC10252364; doi:10.3390/ijerph20115957)
Supplement: Supplementary file 1 [file ijerph-20-05957-s001.zip › ijerph-2331161-supplementary/Table S3.pdf]

Table S3. Mortality for malignant pleural mesothelioma, among males, 2010-2019. Statistically significant clusters (p-value <0.10).

Area	Cluster number	Radius (km)	Number of municipalities	Observed	Expected	RR
NW	1 [a]	9.34	15	205	14.98	14.35
NW	2 [b]	9.85	10	477	191.51	2.68
NW	3 [c]	9.28	9	192	43.13	4.62
NW	4 [d]	9.14	19	79	13.30	6.04
NW	5 [e]	6.50	14	84	40.99	2.07
NW	6 [f]	9.82	9	23	5.89	3.92
NW	7 [g]	2.58	2	12	2.30	5.23
NW	8 [h]	4.93	14	45	21.67	2.09
NE	9 [i]	9.27	16	109	18.28	6.26
NE	10 [j]	5.01	2	117	41.92	2.91
NE	11 [k]	9.41	5	64	32.46	2.01
NE	12 [l]	0	1	54	28.31	1.93
NE	13 [m]	9.62	9	51	26.59	1.94
C	14 [n]	0	1	79	18.21	4.56
C	15 [o]	0	1	36	11.23	3.27
C	16 [p]	8.74	5	44	18.05	2.49
C	17 [q]	9.84	4	20	4.83	4.19
C	18 [r]	8.22	2	20	6.89	2.93
C	19 [s]	9.15	6	22	8.22	2.71
C	20 [t]	0	1	29	12.81	2.29
S	21 [u]	0	1	72	17.08	4.44
S	22 [v]	8.31	2	67	30.59	2.27
S	23 [w]	9.64	13	161	103.25	1.65
S	24 [x]	5.77	3	31	9.85	3.21
S	25 [y]	9.49	7	25	7.54	3.37
S	26 [z]	3.48	2	22	6.81	3.28
SIC	27 [aa]	0	1	16	2.12	7.77
SIC	28 [bb]	8.40	3	20	6.37	3.23

SIC	29 [cc]	0	1	30	12.68	2.45
SAR	30 [dd]	0	1	7	1.2	6.05

[a] Casale Monferrato, Rosignano Monferrato, San Giorgio Monferrato, Frassineto Po, Villanova Monferrato, Morano sul Po, Cella Monte, Coniolo, Terruggia, Ticineto, Balzola, Borgo San Martino, Ozzano Monferrato, Occimiano, Treville.

[b] Genova, Serra Riccò, Sant'Olcese, Busalla, Campomorone, Ceranesi, Davagna, Mignanego, Savignone, Montoggio.

[c] La Spezia, Vezzano Ligure, Arcola, Lerici, Portovenere, Riomaggiore, Santo Stefano di Magra, Riccò del Golfo di Spezia, Follo.

[d] Broni, Stradella, Cigognola, Corvino San Quirico, Pietra de' Giorgi, Barbianello, Golferenzo, Portalbera, Santa Giuletta, Santa Maria della Versa, Zenevredo, Bosnasco, Campospinoso, Canneto Pavese, Casanova Lonati, Mornico Losana, Redavalle, San Cipriano Po, Torricella Verzate.

[e] Legnano, Parabiago, Canegrate, Busto Garolfo, Dairago, Villa Cortese, Inveruno, Buscate, Cuggiono, Mesero, Ossona, Casorezzo, San Giorgio su Legnano, Arconate.

[f] Trino, Cerrina Monferrato, Gabiano, Mombello Monferrato, Palazzolo Vercellese, Pontestura, Odalengo Grande, Ronsecco, Tricerro.

[g] Cengio, Millesimo.

[h] Dalmine, Ponte San Pietro, Treviolo, Mapello, Osio Sopra, Suisio, Bonate Sopra, Filago, Chignolo d'Isola, Terno d'Isola, Bonate Sotto, Curno, Madone, Presezzo.

[i] Monfalcone, Ronchi dei Legionari, Staranzano, San Canzian d'Isonzo, Fogliano Redipuglia, Cervignano del Friuli, San Pier d'Isonzo, Aiello del Friuli, Aquileia, Fiumicello Villa Vicentina, Romans d'Isonzo, Ruda, Doberdò del Lago, Terzo d'Aquileia, Turriaco, Sagrado.

[j] Trieste, Muggia.

[k] Reggio nell'Emilia, Correggio, Bagnolo in Piano, Cadelbosco di Sopra, San Martino in Rio.

[l] Ravenna.

[m] Mira, Spinea, Mirano, Campagna Lupia, Dolo, Pianiga, Stra, Camponogara, Fossò.

[n] Livorno.

[o] Ancona.

[p] Carrara, Massa, Forte dei Marmi, Seravezza, Montignoso.

[q] Rosignano Marittimo, Castellina Marittima, Chianni, Santa Luce.

[r] Civitavecchia, Santa Marinella.

[s] Falconara Marittima, Montemarciano, Agugliano, Polverigi, Monte San Vito, Offagna.

[t] Terni.

[u] Taranto.

[v] Bari, Triggiano.

[w] Napoli, Torre del Greco, Portici, Casalnuovo di Napoli, Ercolano, San Giorgio a Cremano, Volla, Casoria, Sant'Anastasia, Casavatore, Cercola, Massa di Somma, San Sebastiano al Vesuvio.

[x] Pozzuoli, Procida, Bacoli.

[y] Grottaglie, San Giorgio Ionico, Leporano, Carosino, Pulsano, Faggiano, Monteiasi.

[z] Castellammare di Stabia, Gragnano.

[aa] San Cataldo.

[bb] Augusta, Melilli, Priolo Gargallo.

[cc] Siracusa.

[dd] La Maddalena.
