# Supplementary material for: The Epidemiological Surveillance of Mesothelioma Mortality in Italy as a Tool for the Prevention of Asbestos Exposure
Source: Int J Environ Res Public Health. 2023 May 25;20(11):5957. doi: 10.3390/ijerph20115957 (PMC10252364; doi:10.3390/ijerph20115957)
Supplement: Supplementary file 1 [file ijerph-20-05957-s001.zip › ijerph-2331161-supplementary/Table S4.pdf]

Table S4. Mortality for malignant pleural mesothelioma, among females, 2010-2019. Statistically significant clusters (p-value <0.10).

Area	Cluster number	Radius (km)	Number of municipalities	Observed	Expected	RR
NW	1 [a]	9.49	14	168	7.69	23.83
NW	2 [b]	3.44	5	56	3.45	16.70
NW	3 [c]	4.56	6	15	2.87	5.25
NW	4 [d]	0	1	7	0.51	13.87
NE	5 [e]	5.34	4	14	2.94	4.86
NE	6 [f]	6.22	3	9	1.51	6.05
NE	7 [g]	8.42	5	9	1.54	5.92
C	8 [h]	0	1	27	5.81	4.93
S	9 [i]	8.31	4	35	10.37	3.66
S	10 [j]	7.22	13	68	34.50	2.23
S	11 [k]	0	1	21	5.19	4.26
SIC	12 [l]	0	1	9	0.53	18.05

[a] Casale Monferrato, San Giorgio Monferrato, Frassineto Po, Occimiano, Ozzano Monferrato, Pontestura, Terruggia, Ticineto, Villanova Monferrato, Balzola, Rosignano Monferrato, Cella Monte, Motta de' Conti, Treville.

[b] Broni, Stradella, Canneto Pavese, Portalbera, Zenevredo.

[c] Sarnico, Paratico, Credaro, Predore, Viadanica, Villongo.

[d] Calcio.

[e] Monfalcone, Ronchi dei Legionari, Staranzano, Fogliano Redipuglia.

[f] Bagnolo in Piano, Castelnovo di Sotto, Cadelbosco di Sopra.

[g] Castel San Giovanni, Borgonovo Val Tidone, Pianello Val Tidone, Sarmato, Ziano Piacentino.

[h] Livorno.

[i] Bari, Noicattaro, Cellamare, Valenzano.

[j] Napoli, Casoria, Afragola, Giugliano in Campania, Caivano, Calvizzano, Marano di Napoli, Volla, Cardito, Casalnuovo di Napoli, Casavatore, Frattamaggiore, Villaricca.

[k] Taranto.

[l] Biancavilla.
