# Supplementary material for: The Epidemiological Surveillance of Mesothelioma Mortality in Italy as a Tool for the Prevention of Asbestos Exposure
Source: Int J Environ Res Public Health. 2023 May 25;20(11):5957. doi: 10.3390/ijerph20115957 (PMC10252364; doi:10.3390/ijerph20115957)
Supplement: Supplementary file 1 [file ijerph-20-05957-s001.zip › ijerph-2331161-supplementary/Table S6.pdf]

Table S6. Mortality for malignant mesothelioma of peritoneum, among females, 2010-2019. Statistically significant clusters (p-value <0.10).

Area	Cluster number	Radius (km)	Number of municipalities	Observed	Expected	RR
NW	1 [a]	1.83	2	9	0.58	16.97
SIC	2 [b]	6.28	2	3	0.12	33.81

[a] Grugliasco, Collegno

[b] Biancavilla, Belpasso
